# Efficacy of Empiric Antibiotic Coverage in Community-Acquired Pneumonia Associated with Each Atypical Bacteria: A Meta-Analysis

**DOI:** 10.3390/jcm10194321

**Published:** 2021-09-23

**Authors:** Khalid Eljaaly, Ahmed Aljabri, Ali A. Rabaan, Ohoud Aljuhani, Abrar K. Thabit, Mohannad Alshibani, Thamer A. Almangour

**Affiliations:** 1 Department of Pharmacy Practice, Faculty of Pharmacy, King Abdulaziz University, Jeddah 21589, Saudi Arabia; amaljabri@kau.edu.sa (A.A.); oaljuhani@kau.edu.sa (O.A.); akthabit@kau.edu.sa (A.K.T.); malshibani@kau.edu.sa (M.A.); 2 Department of Pharmacy Practice and Science, College of Pharmacy, University of Arizona, Tucson, AZ 85724, USA; 3 Molecular Diagnostic Laboratory, Johns Hopkins Aramco Healthcare, Dhahran 31311, Saudi Arabia; ali.rabaan@jhah.com; 4 Department of Clinical Pharmacy, College of Pharmacy, King Saud University, Riyadh 11451, Saudi Arabia; talmangour@ksu.edu.sa

**Keywords:** atypical bacteria, legionella, mycoplasma, chlamydia, antibiotic

## Abstract

The benefit of empiric coverage for community-acquired pneumonia (CAP) for atypical bacteria is controversial. This meta-analysis purpose was to compare the clinical failure rate between adults who empirically received atypical coverage versus those who did not. We searched PubMed and EMBASE for randomized controlled trials (RCTs), comparing the clinical failure rate of CAP associated with individual atypical bacteria between adults who received empiric atypical coverage versus those who did not. Risk differences (RDs) with 95% confidence intervals (CIs) were calculated using random-effects models. Eight double-blind RCTs (65 patients with *Legionella* spp., 176 patients with *M. pneumoniae*, and 78 patients with *C. pneumoniae*) were included in the meta-analysis. The rate of clinical failure was significantly lower with empiric atypical coverage in CAP associated with *Legionella* spp. (RD, −42.6%; 95% CI, −69.8% to −15.4%; *p*-value = 0.002; I^2^ = 0%) and *Mycoplasma pneumoniae* (RD, −9.5%; 95% CI, −18.9% to −0.1%; *p*-value = 0.048; I^2^ = 0%), but not with *Chlamydia pneumoniae* (RD, 7.1%; 95% CI, −9.0% to 23.1%; *p*-value = 0.390; I^2^ = 0%). This meta-analysis of RCTs found that empiric atypical coverage decreased the clinical failure rate of CAP associated with *Legionella* spp. and *M. pneumoniae*, but not with *C. pneumoniae*.

## 1. Introduction

Community-acquired pneumonia (CAP) is one of the most common infections worldwide and is associated with significant morbidity and mortality [1,2,3]. Major causative pathogens include both typical bacteria, such as *Streptococcus pneumoniae* and *Haemophilus influenzae*, and atypical bacteria, such as *Legionella* spp., *Mycoplasma pneumoniae,* and *Chlamydophila pneumoniae.* The international incidence of atypical bacteria in patients with CAP is estimated to be 22% and varies according to the geographical region. For instance, the incidence of atypical bacteria in the US is approximately 4%, whereas in China, the incidence is approximately 40%, exceeding that of *S. pneumoniae* [4,5]. The true incidence is likely underestimated since it is not a standard of care in many countries to microbiologically identify these pathogens in respiratory cultures especially in the outpatient setting. Considering the undistinguished clinical features between atypical vs. typical pathogens and the lack of an accurate and rapid diagnostic tool for pathogen identification, starting patients with CAP empirically on an antibiotic with atypical coverage might be warranted.

Atypical bacteria are covered by either macrolides, doxycycline, or fluoroquinolones. It is still debatable whether CAP patients must receive empiric antibiotic therapy for atypical bacteria [6]. The potential risk of side effects, drug–drug interactions, microbial resistance, and cost of adding anti-bacterial coverage against atypical bacteria should be weighed versus the risk of worse clinical outcomes with missing this antibacterial activity [6,7,8,9]. Several major guidelines recommended adding empiric atypical coverage for hospitalized patients, particularly those with moderate to high severity [1,2,3]. The finding from a recent meta-analysis of randomized controlled trials (RCTs) supports this recommendation, as significantly more patients clinically failed therapy in the group receiving antibiotics lacking atypical bacterial activity [6]. However, it is unclear which atypical bacteria benefited from the empiric coverage and whether the difference found was due to clinical failure of bacteria other than atypical bacteria. The objective of this meta-analysis was to compare the difference in the rate of clinical failure of CAP associated with each atypical bacteria between adults who empirically received atypical bacterial coverage versus those who did not.

## 2. Materials and Methods

This systematic review and meta-analysis was performed according to the Preferred Reporting Items for Systematic Reviews and Meta-Analyses (PRISMA) guideline.

### 2.1. Search Strategy and Study Selection Criteria

We searched the EMBASE and PubMed bibliographic databases until 21 March 2021. Two authors independently extracted the data and screened the literature without restricting language or date. The search strategy is provided in Appendix A (Table A1). RCTs reporting clinical efficacy of empiric atypical coverage (with fluoroquinolones, macrolides, or doxycycline) versus no coverage (i.e., β-lactams) in adults with CAP caused by individual atypical bacteria (*Legionella* spp., *M. pneumoniae*, and *C. pneumoniae*) were included. RCTs of pediatrics and those not reporting clinical failure of CAP caused by individual atypical bacteria were excluded.

### 2.2. Outcomes, Data Analysis, and Risk of Bias

The study outcome was the rate of clinical failure among each atypical bacteria. Mantel–Haenszel risk differences (RDs) with 95% confidence intervals (CIs) were calculated using random-effects models, and heterogeneity (I^2^) was assessed using Cochran’s Q test. The study quality was assessed via the Cochrane risk of bias tool for RCTs (low, unclear, or high). All analyses were done using the Comprehensive Meta-Analysis v.3 software (Biostat, Englewood, NJ, USA).

## 3. Results

### 3.1. Search Results and Study Characteristics

**Figure 1:**
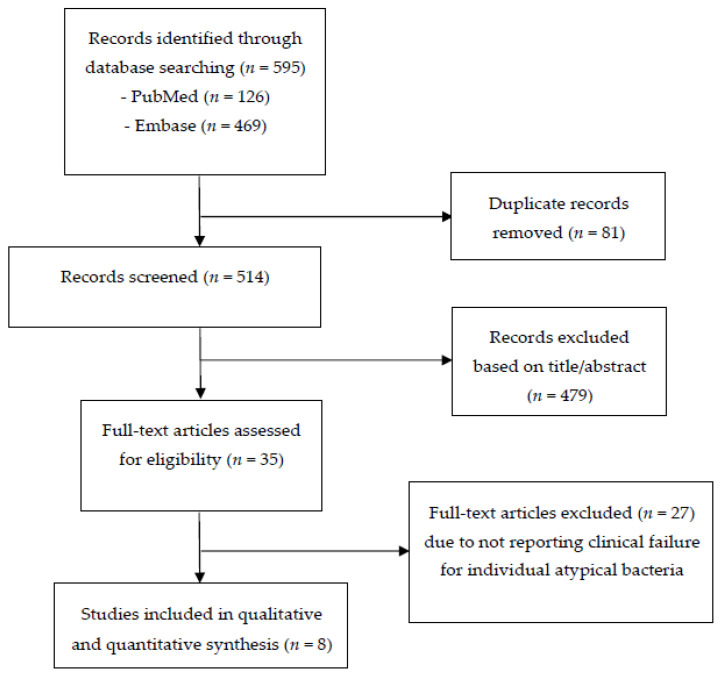
Flow diagram of the study selection process.

The search process revealed 595 articles, and a total of eight RCTs were included eventually as shown in Figure 1. A total of 65 patients with *Legionella* spp., 176 patients with *M. pneumoniae*, and 78 patients with *C. pneumoniae* were included. The diagnosis of atypical bacteria was based on serology. The characteristics of included studies are provided in Table 1, and the study quality assessment is provided in Table 2. All included studies were published [10,11,12,13,14,15,16] except one (Grunenthal 2000; study report KF5501/16). All were multi-centered and double-blinded RCTs. All RCTs were multicontinental (except one), industry-sponsored (except one), and included non-severe CAP (except two included CAP of any severity). The antibiotics used in the empiric atypical coverage arm of all RCTs were fluoroquinolones, but one study also used erythromycin. The duration of antibiotic therapy ranged from 5–14 days.

### 3.2. Study Outcomes

**Figure 2:**
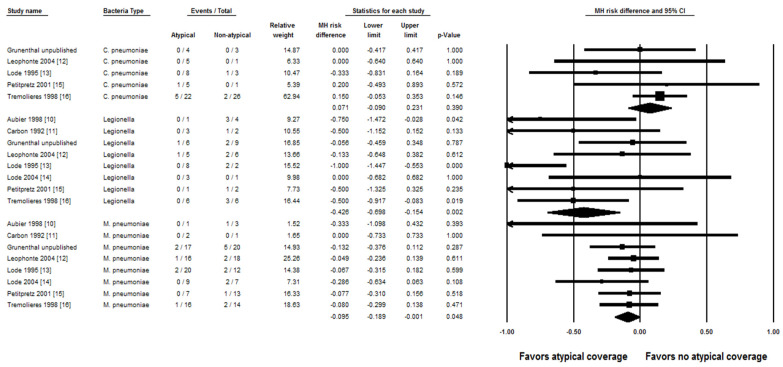
Forest plot showing the risk differences of clinical cure of community-acquired pneumonia associated with each atypical bacteria between adults who empirically received atypical bacterial coverage versus those who did not use random-effects models. Central vertical line, “no difference” point between the two groups; horizontal line, 95% confidence interval; squares, risk differences; diamonds, pooled risk differences. CI, confidence interval; MH, Mantel–Haenszel.

The rate of clinical failure was significantly lower with empiric atypical coverage in patients with *Legionella* spp. (RD, −42.6%; 95% CI, −69.8% to −15.4%; *p*-value = 0.002; I^2^ = 0%) and *M. pneumoniae* (RD, −9.5%; 95% CI, −18.9% to −0.1%; *p*-value = 0.048; I^2^ = 0%) (Figure 2). There was no significant difference in rate of clinical failure in patients with *C. pneumoniae* (RD, 7.1%; 95% CI, −9.0% to 23.1%; *p*-value = 0.390; I^2^ = 0%).

## 4. Discussion

Our meta-analysis main finding is that the rates of clinical failure of treating *Legionella* spp. and *M. pneumoniae* were significantly lower in patients treated with antibiotics with atypical bacterial coverage compared to patients treated with antibiotics lacking atypical bacterial activity. The rate of clinical failure of treating *C. pneumoniae* was not significantly different between the two arms. The antibiotics with atypical coverage used in all the RCTs were fluoroquinolones except for one study, which used erythromycin. Although it was not included as an outcome in our meta-analysis, it is important to consider the adverse reactions of agents used for atypical bacteria. These reactions were assessed in the previous meta-analyses, and it depends on the antibiotic type used to cover atypical bacteria. The inclusion of RCTs improves the internal validity of the meta-analysis. In observational studies, the arm with atypical coverage would likely include the sicker patients in addition to other confounders in non-randomized studies. Moreover, all the included studies were multicenter and multicontinental, which also improves the external validity. Another strength is that all the included RCTs were double-blinded. The majority of the included patients had mild to moderate CAP. Including patients with severe infection could potentially increase the rate of clinical failure in the arm lacking atypical bacterial activity. This is particularly relevant to infections due to *M. pneumoniae* and *C. pneumoniae*, since mild infections are generally self-limiting, and including them could dilute the difference in the rates of clinical failure between the two arms.

Historically, the addition of an antibiotic with atypical coverage to a β-lactam for the management of CAP has been an area of debate. The cluster-RCT by Postma et al. found β-lactams monotherapy to be non-inferior to β-lactams-macrolide combination therapy or fluoroquinolone monotherapy with respect to 90 day mortality in patients admitted to non-intensive care unit wards [17]. This study deviated from the assigned empiric β-lactam monotherapy by allowing addition of empiric coverage of atypical bacteria to β-lactams, and it did not assess the rate of clinical failure. Major current guidelines recommend starting empiric regimens that include atypical coverage [1,2]. Moreover, a recent meta-analysis of RCTs has shown that starting hospitalized CAP patients on guideline-concordant empiric antibiotics with atypical coverage was associated with a significant reduction in the rate of clinical failure [6]. However, it was unknown if the difference in efficacy in this meta-analysis was due to the eradication of atypical bacteria versus other bacteria. The results of our meta-analysis are consistent with the guidelines recommendations and confirmed the benefits of starting empiric atypical coverage for patients with CAP. Some clinicians in some countries may not prefer routine empiric coverage of atypical bacteria in CAP patients, especially in areas with a low incidence of atypical pathogens. They may prefer the individualization of empiric therapy based on risk factors. However, more studies are needed, as limited data are available for risk factors for atypical bacteria. Risk factors for infection with *Legionella* spp. include age > 50 years, diabetes, chronic obstructive pulmonary disease (COPD), smoking, and immunosuppression [18]. For *M. pneumoniae*, young age and crowded settings can increase the risk of contracting this pathogen [19].

The clinical failure rate of treating *C. pneumoniae* was not different between the two arms in this meta-analysis. This result should be interpreted carefully, since patients in the included trials had mild–moderate infection, and studies have shown that mild CAP infection due to *M. pneumoniae* and *C. pneumoniae* in many cases are self-limiting and patients improved spontaneously [20]. Unlike *M. pneumoniae and C. pneumoniae,* CAP due to *Legionella* Spp. is usually more severe and has significant mortality if left untreated [18]. Interestingly, studies of children showed that antibiotics for mild *M. pneumoniae* CAP decreased the morbidity and shortened the symptom duration [21]. However, the Infectious Disease Society of America guidelines for CAP stated that “The evidence to support specific treatment of these microorganisms in adults is lacking” [22].

## 5. Conclusions

Our meta-analysis of double-blind RCTs of adults, mostly with mild to moderate severity of infection, found a significantly lower rate of clinical failure with empiric atypical coverage in CAP associated with *Legionella* spp. and *M. pneumoniae* but not with *C. pneumoniae*. These findings generally support including empiric atypical bacterial coverage for CAP. Future studies should compare fluoroquinolones versus macrolides for CAP due to *Legionella* spp. and *M. pneumoniae*.

## Figures and Tables

**Table 1:** Characteristics of the included studies.

Study	Study Period	Design	Location	Funding Source (Manufactured Drug)	*Legionella* spp.	*M. Pneumoniae*	*C. Pneumoniae*	Characteristics of Community-Acquired Pneumonia	Atypical vs. Non-Atypical Regimen	Duration of Therapy (Days)
Aubier 1998 [10]	1991–1992	Superiority, double-blind, RCT	55 sites in 3 countries (Europe, South Africa)	Non-industry	1 vs. 4	1 vs. 3	NA	Hospitalized, mild–moderate	Sparfloxacin PO 400 mg q24h, then 200 mg q24h vs. amoxicillin 1 g PO q24h	10–14
Carbon 1992 [11]	1989–1990	Superiority, double-blind, RCT	27 sites in France	Industry (temafloxacin)	3 vs. 2	2 vs. 1	NA	Hospitalized, mild–moderate	Temafloxacin 600 mg PO q12h vs. amoxicillin 500 mg PO q8h	10
Leophonte 2004 [12]	1998–1999	Superiority, double-blind, RCT	102 sites in 3 countries (Europe, South Africa)	Industry (gemifloxacin)	5 vs. 6	16 vs. 18	5 vs. 1	>90% hospitalized, suspected pneumococcal mild–moderate	Gemifloxacin 320 mg PO q24h vs. amoxicillin/clav 1.2 g PO q8h	7 vs. 10
Lode 1995 [13]	1990–1992	Superiority, double-blind, RCT	124 sites in 9 countries (Europe, Israel)	Industry (sparfloxacin)	8 vs. 2	20 vs. 12	8 vs. 3	Hospitalized and outpatients, mild–moderate	Sparfloxacin PO 400 mg once, then 200 mg q24h or erythromycin 1 g PO q12h vs. amoxicillin/clav 625 mg PO q8h	7–14
Lode 2004 [14]	1997–1998	Superiority, double-blind, RCT	73 sites in 16 countries (mostly Europe)	Industry (gatifloxacin)	3 vs. 1	9 vs. 7	NA	Hospitalized, mild–moderate	Gatifloxacin 400 mg PO q24h vs. amoxicillin/clav 625 mg PO q8h	5–10
Petitpretz 2001 [15]	1997–1998	Superiority, double-blind, RCT	82 sites in 20 countries (Europe, South America, Australia, Africa)	Industry (moxifloxacin)	1 vs. 2	7 vs. 13	5 vs. 1	79% hospitalized, mild–moderate	Moxifloxacin 400 mg PO q24h vs. amoxicillin 1 g PO q8h	10
Tremolieres 1998 [16]	1995–1996	Superiority, double-blind, RCT	44 sites in Europe, South Africa, Costa Rica	Industry (trovafloxacin)	6 vs. 6	16 vs. 14	22 vs. 26	75% hospitalized, any severity	Trovafloxacin 200 mg PO q24h vs. amoxicillin 1 g PO q8h	7–10
Grunenthal 2000 (unpublished; KF5501/16)	1999–2000	Superiority, double-blind, RCT	132 sites, multinational	Industry (gatifloxacin)	6 vs. 9	17 vs. 20	4 vs. 3	Hospitalized, any severity	Gatifloxacin 400 mg PO q24h vs. amoxicillin 1 g PO q8h	7–10

**Table 2:** Quality assessment of the included studies.

	Selection Bias		Performance Bias	Detection Bias	Attrition Bias	Reporting Bias	Other Bias
Study	Random Sequence Generation	Allocation Concealment	Blinding of Participants and Personnel	Blinding of Outcome Assessment	Incomplete Outcome Data	Selective Reporting	Other Bias
Aubier 1998 [10]	?	?	+	+	−	+	+
Carbon 1992 [11]	?	?	+	+	−	+	−
Leophonte 2004 [12]	?	?	+	+	−	−	?
Lode 1995 [13]	?	?	+	+	+	+	?
Lode 2004 [14]	+	+	+	+	+	+	?
Petitpretz 2001 [15]	+	+	+	+	−	+	?
Tremolieres 1998 [16]	?	?	+	+	+	+	?
Grunenthal 2000 (unpublished; KF5501/16)	?	?	+	+	+	+	?

+: low risk of bias, ?: unclear risk of bias, −: high risk of bias.

## Data Availability

Not applicable.

## Appendix A

**Table A1:** Search Strategy.

Database/Search Dates	Search Strategy
PubMed/MEDLINE, National Library of Medicine (Searched until 21 March 2021)	#1-“Macrolides”[Mesh] OR “Fluoroquinolones”[Mesh] OR
	“Doxycycline”[Mesh]
	#2-“beta-Lactams”[Mesh]
	#3-pneumonia[Mesh]
	#4-#1 AND #2 AND #3 AND “randomized controlled trial”[Publication Type]
Embase, Elsevier (Searched until 21 March 2021)	#1-‘macrolide’/exp OR ‘quinoline derived antiinfective agent’/exp OR
	‘doxycycline’/exp
	#2-‘beta lactam antibiotic’/exp
	#3-‘pneumonia’/exp
	#4-#1 AND #2 AND #3 AND [randomized controlled trial]/lim
